# Temporal and Spectral Models as Correlates to Auditory-Perceptual Judgments of Overall Severity and Listener Comfort in Tracheoesophageal Voice

**DOI:** 10.3390/app14010214

**Published:** 2023-12-26

**Authors:** Philip C. Doyle, Hamzeh Ghasemzadeh, Jeff Searl

**Affiliations:** 1 Otolaryngology Head and Neck Surgery, Division of Laryngology, School of Medicine Stanford University, Stanford University, Stanford, CA 94305, USA; 2 Center for Laryngeal Surgery and Voice Rehabilitation, Massachusetts General Hospital, Boston, MA 02114, USA; 3 Department of Surgery Harvard Medical School, Boston, MA 02114, USA; 4 Department of Communicative Sciences and Disorders, Michigan State University, East Lansing, MI 48824, USA

**Keywords:** tracheoesophageal speech, auditory-perceptual evaluation, voice disorders, perceptual psychophysics

## Abstract

Introduction: This study pursued two objectives: (1) to determine the potential association between listener (*n* = 51) judgments of 20 male tracheoesophageal speaker samples for two auditory-perceptual dimensions of voice, overall severity (OS) and listener comfort (LC); and (2) to assess the temporal and spectral acoustic correlates for these auditory-perceptual dimensions. Methodology: Three separate correlation analyses were performed to evaluate the association between OS and LC. First, scores of OS and LC from all listeners were pooled together, and then the correlation between OS and LC was computed. Second, scores of OS and LC were averaged over all listeners to derive a single estimate of OS and LC for each TE speaker sample; the correlation between the average OS and LC was then computed. Third, listener-to-listener variability in the association between OS and LC was evaluated by computing the correlation between OS and LC scores from each listener across all TE samples. Finally, two stepwise multiple regression models were created to relate the average LC score to spectral and temporal variation in the acoustic signal. Results: While the pooled OS and LC scores had a moderate positive correlation (r = 0.66, *p* < 0.00001), the averaged OS and LC exhibited a near perfect positive correlation (r = 0.99, *p* < 0.00001). The significant differences between the pooled and averaged scores were explained by significant listener-to-listener variability in the association between OS and LC. OS and LC scores from 5 listeners had non-significant correlations, 10 had moderate correlations (r < 0.7), 35 listeners had high correlations (0.7 < r < 0.9), and 1 listener had a very high correlation (r < 0.9 < 1). Finally, the acoustic models created based on the spectral and temporal variations in the signal were able to account for 87.7% and 61.8% of variation in the average LC score. Conclusions: The strong correlations between OS and LC suggest that LC may, in fact, provide a more comprehensive auditory-perceptual surrogate for the voice quality of TE speakers. Although OS and LC are distinct conceptual dimensions, LC appears to have the advantage of assessing the social impact and potential communication disability that may exist in interactions between TE speakers and listeners.

## 1. Introduction

In 1980, Singer and Blom presented the first report that detailed the surgical-prosthetic method of tracheoesophageal (TE) puncture voice restoration in 60 individuals who had undergone total laryngectomy for laryngeal cancer [1]. Over the interceding 40+ years since the introduction of this postlaryngectomy voicing option, the clinical literature has been replete with reports on its widespread use and success as an alaryngeal voice rehabilitation ([2–5] and others). Overall, this postlaryngectomy, alaryngeal method of voice and speech has gained wide acceptance as a rehabilitation option. Both empirical information on TE speech as well as clinical reports suggest a range of acoustic advantages that cross the frequency, intensity, and temporal domains associated with voice and speech production [6,7]. Further, TE voice restoration has been shown to exhibit relatively good levels of speech intelligibility compared with other alaryngeal methods such as esophageal speech and the use of the electrolarynx [7]. However, despite the apparent success of TE puncture voice restoration as an alaryngeal method, TE speech remains perceptually “abnormal” in comparison to normal laryngeal speakers; additionally, intrinsic variability between speakers clearly exists, and this type of variability directly influences auditory-perceptual judgments by listeners [8–12].

Current data indicate that factors inherent to the TE voice signal may affect listener judgments and that these factors are multidimensional in nature [13]. This is primarily accounted for by the nature of the postlaryngectomy voicing source and its interaction with the aerodynamic driving source (i.e., the lungs) during TE voice production. Thus, TE speech is typically characterized by reduced and variable pitch, inconsistent vocal intensity, and variable degrees of aperiodic vocal noise (i.e., “roughness”) that is easily identified by listeners. While both TE speech and traditional esophageal speech utilize the tissues of the lower pharynx and the upper esophagus, or what is commonly termed the pharyngoesophageal segment (PES), as a postlaryngectomy voicing source, TE speech is augmented by access to pulmonary air which serves as the driving source for this alaryngeal method. Therefore, the PES in TE speakers is not an adductor–abductor mechanism, but rather, a postlaryngectomy voicing source that responds to variable airflow through this anatomical voicing conduit.

Even though the initiation of TE voice production and its termination are volitional acts, the PES segment does respond passively to airflow during the process of speech production. Additionally, the PES itself may be quite variable in both its physical mass and its associated compliance and/or resistance to an aerodynamic driving source [14–16]. When this is considered relative to high volumes of pulmonary air, the variability in how the PES responds to airflow (i.e., PES segment vibration) and the resultant voicing signal that is generated will be characterized by a range of variability. As noted, this variability has most commonly been associated with high levels of aperiodicity in the TE signal, an inconsistency that influences both the frequency and intensity of the signal, with a direct impact on signal-to-noise ratios (SNRs). In assessing such findings from the literature in a collective fashion, it is increasingly clear that the composite TE voice signal is complex and inconsistent; further, this complexity can be detected by the listener.

As noted, the TE voice and speech signal is clearly and consistently identified as being abnormal by listeners with several reports documenting that it is judged to be unnatural with varied assessments of its overall acceptability to the listener [17–20]. Thus, the degree of abnormality that is common to TE voice and speech, or perhaps better stated as the degree of *severity* in the composite quality of the TE signal, is expected to have an impact on listeners. Based on the existing literature, we believe that TE voice and speech signals that are increasingly aberrant in their acoustic properties directly influence how comfortable a listener will be in interacting with such speakers [21].

Stated more simply, we believe that as the overall quality of a TE speaker’s voice becomes increasingly poor or its level of deviance from that of a normal speaker increases in severity, this is mirrored in judgments by listeners about how comfortable they would be in interacting socially with such speakers. The assumed link would be two-fold. First, we posit that TE voices that are relatively less abnormal in the perceived degree of severity are also more likely to be less variable based on acoustic characteristics. Accordingly, as acoustic measures become increasingly aberrant, judgments of severity increase commensurately [22]. Secondly, we also believe that as voice quality is judged to be more severely abnormal, listeners become less favorable in their desire or willingness to directly communicate with that TE speaker; hence, their assessments of how comfortable they would be in such interactions are less positive.

Although considerable study of TE speech has been performed relative to the acoustic properties and speech intelligibility ([17,21,23,24] and others), questions regarding how the composite TE voice signal is perceived by listeners remain. That is, the interactions between the overlapped, real-time changes in frequency, intensity, and the temporal pattern of continuous speech will directly influence how a listener evaluates the overall quality and perceived “favorability” of the TE voice signal. At present, little work has addressed the acoustic properties of TE speech in relation to auditory-perceptual assessments of the signal provided by listeners. Thus, we believe that studies that are designed to evaluate auditory-perceptual judgments of TE voice and speech and simultaneously seek to quantify the acoustic properties (e.g., both spectral and temporal) of the TE signal hold considerable merit in enhancing our understanding of this important relationship [22]. Consequently, the purpose of the present study was to assess two specific objectives: (1) to determine the potential relationship between the auditory-perceptual ratings of two perceptual dimensions, overall severity (OS) and listener comfort (LC), in voice samples provided by TE speakers; and (2) to assess the inherent acoustic properties of the TE voice signal in relation to listener judgments of LC through application of spectral and temporal models of analysis.

## 2. Methods

Participant Speakers: The participant speakers included 20 male adults who had undergone total laryngectomy and TE puncture voice restoration (mean age = 62.6 years age, range 57 to 71); all were at least 12 months postlaryngectomy and had successfully used TE speech as their primary method of verbal communication for at least 12 months post-TE puncture at the time of speech sample collection. All participant speakers were native English speakers and none had used TE speech for longer than 8 years at the time of voice sample recording. Prior to their participation, all the speakers had passed a bilateral, pure-tone audiometric hearing screening at ≤30 dBHL for the octave frequencies 250–2000 Hz. Prior to consideration as potential participant speakers, all were judged by two independent raters who were familiar with this method of postlaryngectomy voice and speech rehabilitation to exhibit good-to-excellent levels of speech intelligibility as we desired to reduce any potential influence of poor speech intelligibility on assessments of OS and LC. Prior to obtaining voice sample recordings, all the participant speakers provided their informed consent as per the regulations of the research ethics board specific to this project.

Participant Listeners: Fifty-one normal-hearing young adults (46 females, 5 males; mean age = 22.3 years; range = 20; 9 years to 31; 1 years) served as listeners; all participant listeners were considered to be naïve based on the fact that none had any formal education about voice disorders or postlaryngectomy voice and speech rehabilitation, nor had they had prior exposure to this clinical population. Listeners were native English speakers and all indicated that they had no history of speech, language, and/or hearing difficulties prior to their participation in the study. All listeners passed a pure-tone hearing screening at 20 dB for the octave frequencies 500, 1000, and 2000 Hz prior to their participation. Potential participant listeners who were less than 20 years of age or older than 35 years of age, or those who failed the hearing screening were excluded from consideration. Prior to their participation in the auditory-perceptual phase, all the participant speakers provided their informed consent as per the regulations of the research ethics board.

Auditory-Perceptual Stimuli and Stimulus Preparation: Each speaker recorded the experimental stimuli used in this study as part of a larger, standard voice sample recording protocol. All speaker samples were recorded digitally at a sampling rate of 44 KHz using *SonaSpeech II* (Kay Pentax, Montvale, NJ, USA) under identical conditions in a professional recording environment that was free of ambient noise. Participant speakers represented a range of TE voice qualities and demonstrated substantial interspeaker variability to increase the likelihood that each perceptual dimension under study would be scaled along the breadth of its psychophysical continuum during the psychophysical experimental rating task [25,26].

For the auditory-perceptual phase of the study, the second sentence of the Rainbow Passage [27] was extracted (i.e., “*The rainbow is a division of white light into many beautiful colors*”). Once these sentences were extracted, the experimental listening task was constructed to consist of 25 speech samples; these stimuli represented 20 original speaker samples plus five duplicated samples which were included to assess both intra- and inter-rater agreement.

Listening Procedure. Two auditory-perceptual dimensions, OS and LO, were evaluated in this study. The order of speaker samples presented in all listening tasks (i.e., for both assessments of OS and LO), including the reliability samples, was randomized for both perceptual dimensions and for every participant listener. Ratings of the two dimensions were separated by 5–7 days to control for potential learning effects; thus, OS and LO were presented in a counterbalanced manner across two sessions. All listening sessions were conducted in a quiet, sound-treated listening laboratory that was free of extraneous noise. All digital voice stimuli were presented to listeners via a desktop computer (Dell, Round Rock, TX, USA) and stereo headphones (Sony MDRV-150). All the samples were presented at a comfortable listening level determined by each listener during both sessions.

In making judgments of OS, the listeners were provided with a written definition indicating that this dimension represented “*a comprehensive measure of how ‘good’ or ‘poor’ the voice sample is judged to be*” by the listener [19]. In contrast, for LC, listeners were provided a written definition that asked, “*How comfortable would you feel listening to the person’s speech in a social situation? Your rating should reflect your feelings about the way the person was speaking, not what the person was saying or how their personality affected you*” [28]. Both auditory-perceptual dimensions were rated using a 100-point visual analogue scale (VAS) that was provided on paper. Dependent upon which dimension was being evaluated, listeners were provided with anchors of “normal” and “maximally severe” for OS, and “extremely comfortable” and “extremely uncomfortable” for LC. All scaled ratings by listeners were obtained by asking the listener to listen to each speaker sample and then mark a perpendicular line through a 100 mm (VAS) that represented either the OS or LC dimension. Listeners were permitted to listen to each sample as many times as they wished to prior to making their scaled judgment; however, they were not allowed to return to or listen to any prior sample and/or make changes to the scaled rating provided previously. Listeners evaluated each TE speech sample for both OS and LC over two sessions separated by 5–7 days.

## 3. Data Analysis

Once all participant listeners had completed both listening sessions, the scales for each speaker sample were hand measured in mm and scored from the far left of each scale; this resulted in scores that ranged from 1 to 100 in all cases. For both OS and LC, lower scaled scores represented auditory-perceptual judgments of less OS or more favorable LC based on the definitions and anchors provided for each scale. These scaled scores for each listener were then submitted for further analyses as per the objectives of the study.

Objective 1: Three different correlation analyses were used to quantify the association between OS and LC. In the first, the OS and LC scores from all listeners were pooled. The second analysis investigated the association between OS and LC when speaker scores were averaged across all listeners. The third investigated listener-to-listener variability in the association between OS and LC.

Objective 2: Image representation was used to compute acoustic measures. This approach converted the spectrogram of a speaker’s voice sample into an image and then computed two statistical models and variations in the (a) time, and (b) frequency domains [29]. The *temporal model* reflects the probability of changes in the energy of a fixed sub-band between consecutive time segments (i.e., changes in the *x*-direction), whereas the *spectral model* reflects the probability of changes in the energy of consecutive sub-bands of a spectrogram at a fixed time segment (i.e., changes in the *y*-direction).

Two separate stepwise multiple linear regression analyses were used to explain the perceptual score of LC in terms of acoustic measures of TE speech. The outcome variable in both analyses was the average value of speaker judgments; the predictor variables were the inertia and the sum of rows of the temporal model for the first analysis, and the inertia and the sum of rows of the spectral model for the second. The inertia of a row quantifies the distribution of the probability of changes in the spectrogram (e.g., a low-energy region in a spectrogram followed by a high-energy region). The sum of a row quantifies the probability of different energy levels in the spectrogram (e.g., whether the spectrogram contains concentrated energy bands or the energy is dispersed).

## 4. Results

**Figure 1.**
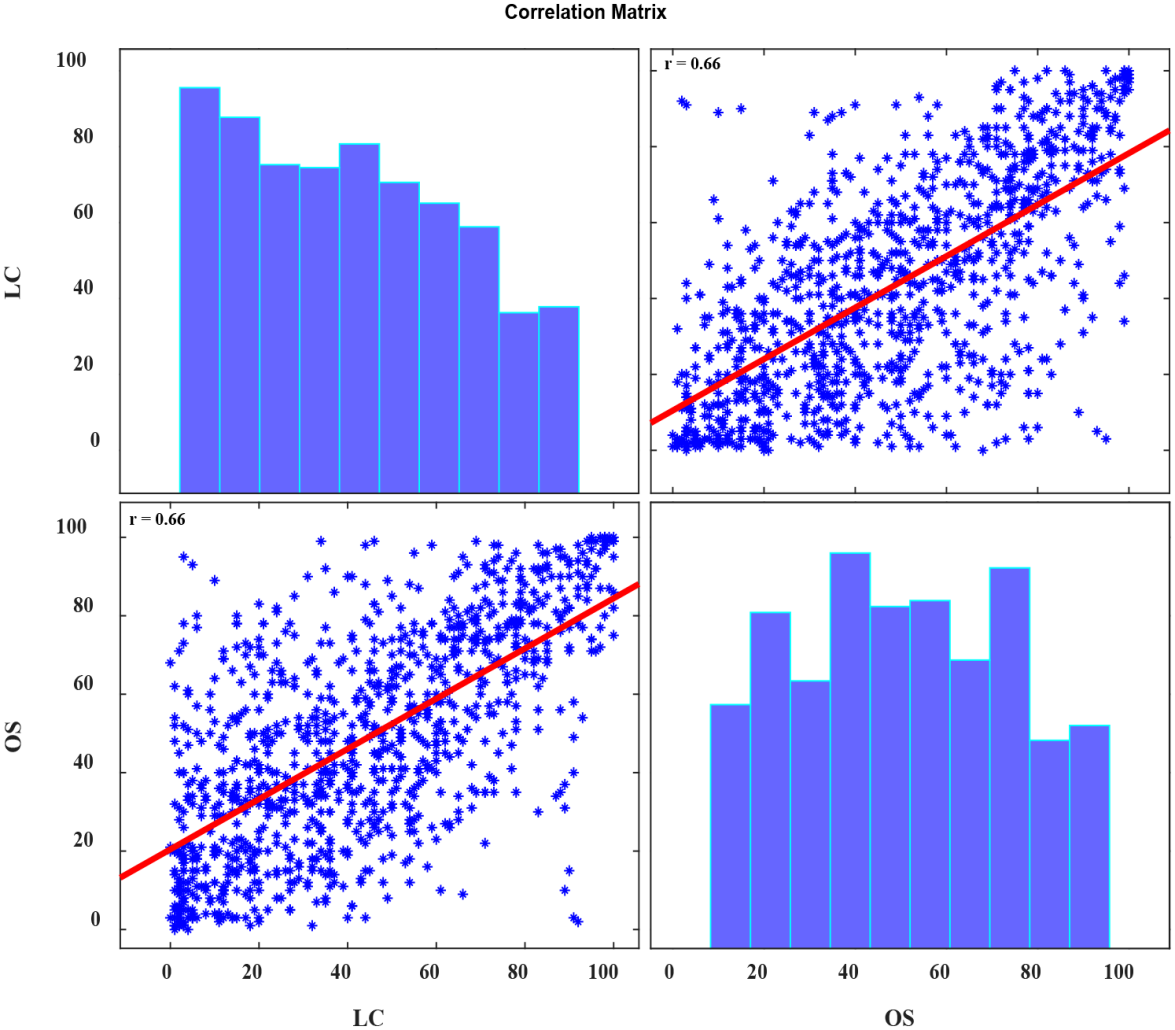
A graphic representation of the association between the perceptual scores of OS and LC when pooled across all listeners. The * symbols represent individual data points and the red line is the line of best fit associated to these data.

First, the scores of OS and LC from all listeners were pooled. The OS scores ranged from 1 to 100 (mean ± std = 48.0 ± 27.0); similarly, the LC scores across speakers ranged from 1 to 100 (mean ± std = 43.0 ± 27.8). The association between the pooled scores was then evaluated using Pearson’s correlation. The two scales had a moderate and positive correlation (r = 0.66, *p* < 0.00001). The results of this pooled data analysis are presented in Figure 1.

**Figure 2.**
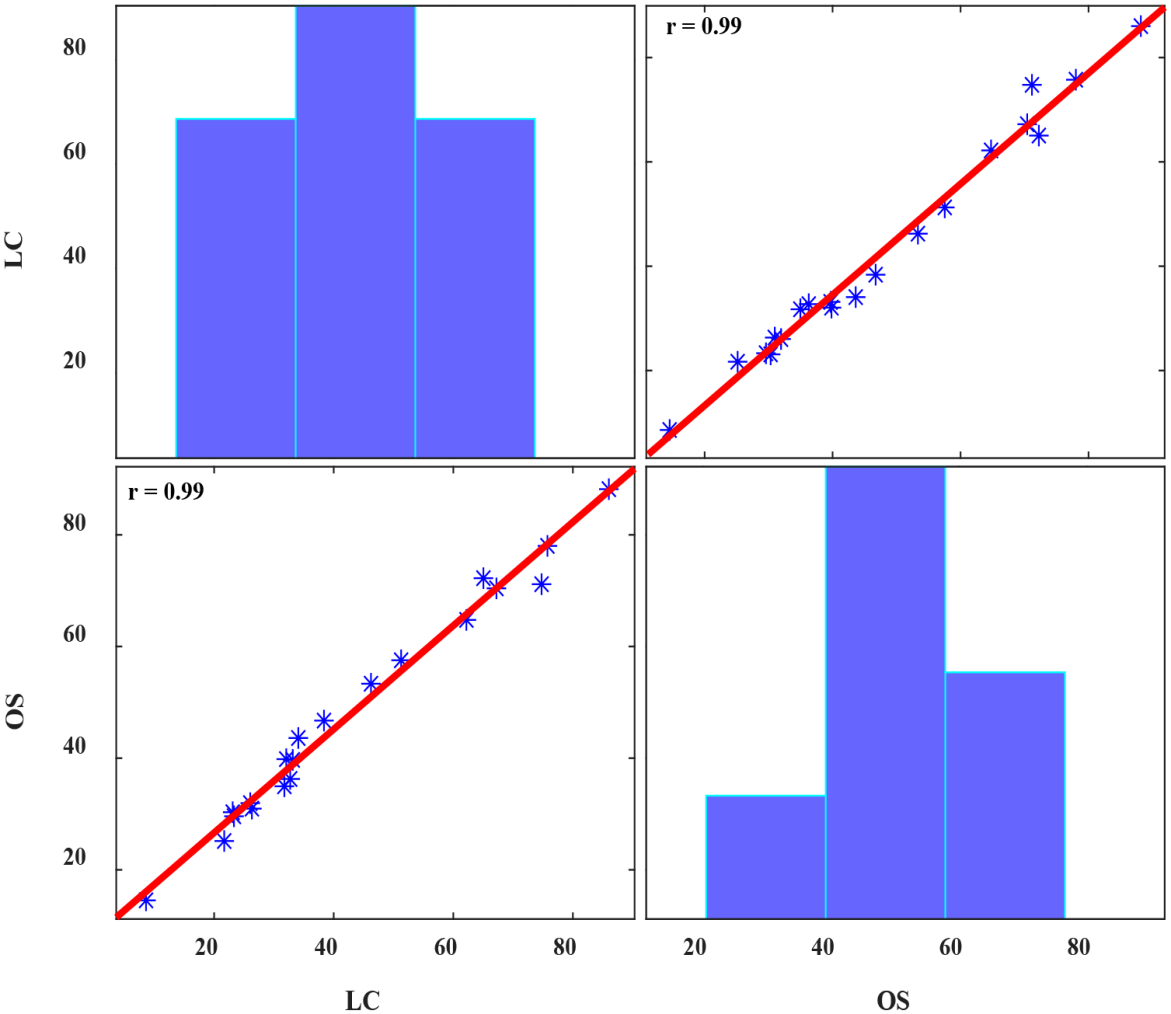
The association between the perceptual scores of OS and LC when averaged across all listeners. The * symbols represent individual data points and the red line is the line of best fit associated to these data.

Second, the scores of OS and LC were averaged over listeners. The average LC scores across speakers ranged from 8.6 to 86 (mean ± std = 43.0 ± 21.7); the OS scores ranged from 14.5 to 88.2 (mean ± std = 48.0 ± 20.3). The association between the average scores was evaluated using Pearson’s correlation. The two scales exhibited an almost perfect correlation (r = 0.99, *p* < 0.00001). Figure 2 provides the graphic results of this analysis. Based on this statistical analysis, our data would appear to provide evidence that these two perceptual measures are likely to evaluate similar underlying auditory-perceptual characteristics inherent to the TE speaker samples investigated in this study.

**Figure 3.**
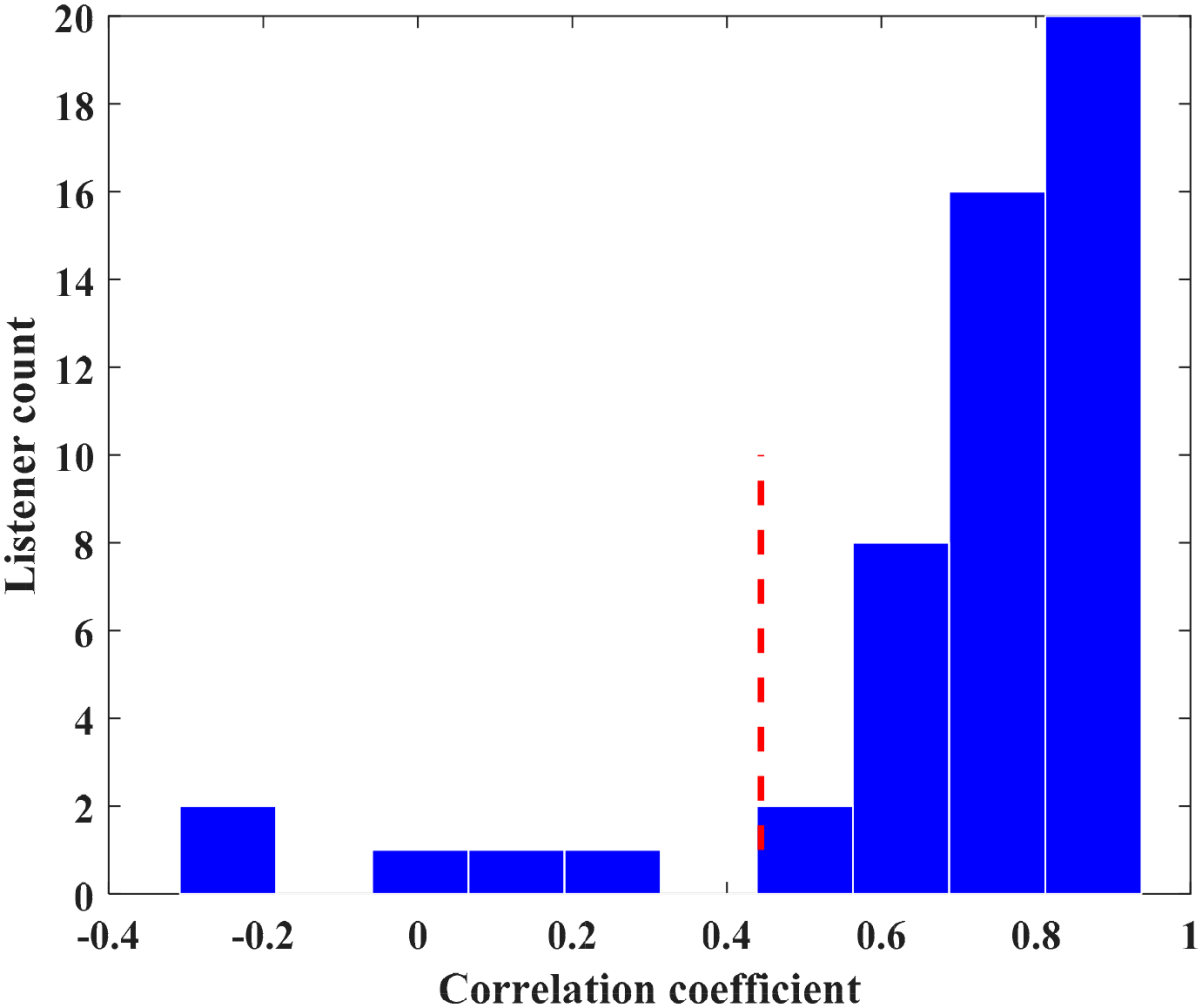
The distribution of correlation coefficients for individual listeners indicates a wide range of listener-to-listener variability between the two dimensions of OS and LC. The dashed red line shows the threshold for a significant correlation.

To determine the possible source of differences between the results of Figures 1 and 2, we investigated listener-to-listener variability in the association between OS and LC. To that end, the correlation coefficient between OS and LC over all tokens (TE samples) was computed separately for each listener. Listeners showed a wide range of variability in the association between OS and LC. Five listeners had non-significant correlations, 10 had moderate correlations (r < 0.7), 35 listeners had high correlations (0.7 < r < 0.9), and 1 listener had a very high correlation (r < 0.9 < 1). Figure 3 depicts the histogram of listener-to-listener variabilities in the association between OS and LC. The intra-judge scores may explain some of the individual variability we observed in the correlation between OS and LC.

In order to further evaluate our data, separate multiple linear regression models were created to explain the average LC scores in terms of spectral and temporal variations of TE speech. The models created based on the spectral and temporal measures of speech accounted for 87.7% and 61.8% of variability in average LC, respectively. The spectral model included the main effect of the inertia of row 11, the sum of row 6, and an interaction effect between the inertia of row 13 and the sum of the last row of the model. All three terms were significant (*p* < 0.05). Generally speaking, patients with higher LC scores were more likely to show more limited spectral variations in the portion of the spectrogram that had moderate energy concentration (i.e., the middle rows of the model). The temporal model was based on three predictors, all of which were significant (*p* < 0.05). The included predictors were the sum of the last row and the inertia of rows 2 and 12. Generally speaking, patients with higher LC scores were more likely to have very bright regions (i.e., spectro-temporal regions with the highest energy concentration) in their spectrogram. Also, they were more likely to show a wide range of temporal variation in the portion of the spectrogram that had moderate energy concentration (i.e., the middle rows of the model).

## 5. Discussion

This study identified relationships between OS and LC for TE speakers and two specific objectives were explored. First, we determined the association between listener judgments of two auditory-perceptual dimensions, overall severity (OS) and listener comfort (LC). These dimensions were assessed by 51 naïve listeners for voice samples provided by 20 postlaryngectomy TE speakers. Second, we explored the utility of temporal and spectral acoustic models as correlates to the auditory-perceptual data. Thus, this study sought to address the auditory-perceptual evaluation of TE speech for two dimensions—OS and LC—as well as explore acoustic models to further quantify the TE samples studied. Our desire was to integrate and explain larger potential relationships between psychophysical assessments of TE voice by listeners and the acoustic characteristics of those speaker samples.

Based on the data obtained, OS and LC are highly correlated. While the definitional boundaries of both OS and LC as provided in this study may overlap to some degree, strict definitions of both dimensions suggest a logical relationship between the two. That is, as judgments of severity related to the voice quality of TE speakers become poorer, listeners also indicate that they become less favorable in their desire to interact with those speakers (based on their voice quality). This finding suggests that listeners may become increasingly uncomfortable as a speaker’s voice quality becomes less normal. While this is not unexpected, we would suggest that the social penalty is strongly associated with the perceived degree of voice abnormality exhibited by a non-normal speaker. These collective data suggest that acoustic assessments which are based on predictive models may provide valuable information in understanding the potential relationship between a TE speaker and how a listener judges such a sample. This is of particular interest with samples that may be judged as grossly abnormal related to the normal voice, those that are characterized by substantial and variable levels of noise (i.e., frequency and/or intensity perturbation), or in situations where the signal-to-noise ratio is degraded.

Although the present data are very specifically tied to postlaryngectomy speakers who had undergone TE voice restoration, it is possible that similar findings could be found with other clinical voice populations. However, while we would anticipate that the transition of these data to other classes of disordered voice may be similar in other voice disordered groups (e.g., those with a dysarthris), one would carefully need to consider the potential influence of other speech or voice factors (e.g., changes in speech rate, articulatory precision, etc.) on judgments of both OS and LC. Therefore, regardless of the underlying cause or class of a voice disorder, changes in one’s overall voice quality and its level of perceived severity relative to normal expectation [25] would likely create reduced levels of listener comfort during communication exchanges and this would include considerations of speaker gender [12,28,30,31]. Finally, it is equally clear that other acoustic models beyond those presently employed, or variations thereof, may provide unique solutions to understanding how a listener perceives and quantifies the TE voice, regardless of the auditory-perceptual dimension(s) under evaluation.

The potential relationship between OS and LC as derived from this empirical project suggests that as voice quality becomes more deviant and, hence, more severely disordered relative to normal expectations, a social penalty will also emerge. Though we did not assess social penalty in the present work, we believe that as a listener’s comfort level during verbal communication is decreased, the likelihood of a potential social penalty is almost certainly increased [8,10,28,31]. When assessing the definitions of both OS and LC used in the present study, we may anticipate that LC captures both a valid index of vocal quality for TE speakers and how a listener may respond to such aberrant voice qualities. It can also be noted that while LC was originally reported as an assessment method to index listener response to those who stutter [28,32], it is clear that this dimension also may address how communicative dyads may be impacted by voice qualities that deviate from normal. Consequently, we would suggest that the influence of voice quality on social interactions must be considered, particularly in the context of rehabilitation efforts.

Accordingly, given the past use of LC in those who stutter [28] and currently in those who use postlaryngectomy TE voice, the possibility of extending the use of LC as a clinical metric to other clinical populations who present with speech and/or voice disorders is warranted. However, we would caution that extreme care must be taken in designing future projects to address and potentially control myriad variables that can directly influence listener judgments of voice and speech [25]. We would also acknowledge that this challenge becomes increasingly more difficult if the accuracy or intelligibility of speech is concomitantly influenced.

The collective data from this study may represent auditory-perceptual dimensions that could be used to document outcome differences across TE speakers. These data also have potential application in assessments and/or comparisons with other alaryngeal speech modes (i.e., esophageal speech and electrolaryngeal speech). Thus, efforts that seek to integrate complex acoustic data to determine potential relationships between TE voice signals and psychophysical assessments may hold considerable promise. Although the present work has exploited spectral and temporal models for this purpose, other approaches to acoustic analysis that seek to identify a concentration or dispersion of signal energy may offer additional insights. Further, because TE voice signals are often characterized by substantial noise, acoustic analyses that seek to quantify multiple types of signal energy can be of value to understanding how listeners respond to such vocal signals. From a comparative point of view, LC may represent an extremely valuable auditory-perceptual dimension in that it may serve to quantify the potential impact of abnormal voice quality and its relationship to social penalty and associated voice-related disability [28,32]. Of particular importance to the present work is the fact that both dimensions assessed herein appear to have strong face validity from the standpoint of psychophysical assessment of voice. However, as noted, LC may serve to capture the larger impact of “severity” secondary to a voice quality disturbance by integrating the listener’s perceived reaction to the voice and their desire or willingness to engage with that speaker [33,34].

Finally, in the context of the TE voice samples studied, we believe that LC has additional value given the range of social stigma that may be associated with laryngeal cancer as a disease, as well as stigma related to its treatment, due to factors such as physical disfigurement, potential causal factors underlying the cancer diagnosis, etc. [10]. Yet, we would caution that the present data cannot be fully generalized to other TE speakers, or to other groups of postlaryngectomy alaryngeal speakers, prior to further investigation. Nevertheless, we believe that the present dataset provides strong evidence that supports the use of LC (and potentially OS) as a simple, yet very sensitive, index of voice quality for TE speakers as part of larger rehabilitation efforts.

## 6. Limitations of the Present Study

Although we believe that our dataset is robust and that the relationships between OS and LC are strong based on our analyses, there are limitations to this work. First, the speaker sample is relatively small (*n* = 20) and the representativeness of these speakers may be questioned. It must be noted, however, that we sought to select a group of TE speakers who exhibited a range of voice qualities while at the same time demonstrating good-to-excellent speech intelligibility. Thus, while we attempted to assess a range of TE voice qualities that would not be directly influenced by the listeners’ ability to understand the content of the speech signal, changes that may occur in voice quality are likely to further influence assessments of both OS and LC. This concern regarding speakers is, however, somewhat offset by a relatively large listener pool (*n* = 51) compared with other auditory-perceptual studies reported in the literature. As a general rule, the size of our listener pool is considerably larger than what typically has been reported in the literature for auditory-perceptual evaluations of TE speakers.

Both dimensions that were studied in this project were operationally defined for the listeners. Nevertheless, we do believe that the complexities associated with grossly unusual or unnatural voice signals such as those common to those who use TE voice and speech will have additive effects on how such signals are perceived and judged by listeners. More directly, it is currently unknown if there is a key or more prominent component of a TE signal that may carry more significant perceptual “weight” relative to judgments of individual speakers. Thus, how any given component of a signal, including changes in frequency and intensity and associated variability (perturbation), the related variations in the relative SNRs and their consistency or inconsistency, alterations in the temporal flow of speech (i.e., speech rate, pause time, linguistic juncture, etc.), are perceptually summed together must be considered.

Currently, there are no data in the literature to suggest that a particular component of a TE voice signal carries more perceptual salience relative to other features that characterize any given sample. Clearly, this type of investigation is worthy of future study. This also raises the question of the potential influence of listener sophistication on the perceptual judgments provided [35] as more experienced listeners may exhibit unique bias relative to their judgments [19,25,30,31]. The other question that may be raised concerning our listener data is related to the fact that the majority of our listeners were women. There is anecdotal evidence to suggest that auditory-perceptual judgments of women and men may not differ for assessments of male TE speakers. However, listener gender must be considered as a relevant potential independent variable when mixed samples that include both male and female speakers are assessed by listeners. This is particularly true for auditory-perceptual judgements that are made with or without prior knowledge of the TE speaker’s gender. With an increasing number of women undergoing laryngectomy, such concerns provide a very important area for future study.

Finally, and in the context of the prior information and speaker gender, it is critical to note that the present data cannot be generalized to women who undergo TE puncture voice restoration. Although women who are laryngectomized and use TE speech will also exhibit a range of variation that crosses the frequency, intensity, and temporal domains of voice and speech production [30,31], they also may experience greater levels of social penalty specific to both the OS and LC dimensions. This is of particular importance should the gender of the speaker be identified to the listener [12]. Past research has shown that women TE speakers may incur less positive listener responses and judgments relative to their male counterparts. In fact, at times, women who use intrinsic methods of alaryngeal voice and speech (e.g., esophageal and TE methods) are often incorrectly identified when the speaker’s gender is unknown [12,30,31,36,37]. For that reason, explorations that actively consider speaker gender as a potential independent experimental variable in future studies would be of substantial benefit.

## 7. Implications of the Data

The literature on postlaryngectomy voice and speech rehabilitation is replete with concerns that there is no widely accepted protocol for assessing a range of characteristics associated with TE speech or other alaryngeal methods. Accordingly, the ability to identify a specific auditory-perceptual dimension that might serve as the “best” or most “comprehensive” indicator of TE voice and its relationship to acoustic models would benefit clinical efforts to document outcomes. Though early in the process, the present work may support and validate the use of LC as a measure of TE puncture voice restoration. However, we would again caution that speech intelligibility must be factored into larger scale use of auditory-perceptual methods of evaluation for all postlaryngectomy speakers regardless of speech mode. Further, and as mentioned previously, how any given component of the TE voice signal is weighted, either favorably or unfavorably, in the context of other features inherent to that sample must always be carefully considered. This must also be considered within the context of how the speaker’s new alaryngeal voice influences social functioning and interpersonal interaction [38–40]. With this concern in mind, our acoustic method may serve to distinguish TE speakers and provide for improved classification methods when coupled with auditory-perceptual data provided by listeners [21].

In summary, our data support the use of acoustic models that address temporal and spectral aspects of the vocal signal in the context of auditory-perceptual judgments. Consequently, questions related to the combined psychophysical and acoustic assessments of TE speech appear to be fruitful avenues of follow-up research. These findings suggest that LC or OS provide a valuable metric of voice/speech performance following TE puncture voice restoration. When the collective results of this work are evaluated, we believe that LC can be utilized as a valuable index of TE speaker performance in both experimental and clinical protocols.

## 8. Conclusions

This study explored the use of two auditory-perceptual dimensions, OS and LC, in a group of postlaryngectomy TE speakers. Listener data were assessed in the context of acoustic measures that were based on temporal and spectral models of analysis. Our findings indicated that while pooled OS and LC scores resulted in a moderate positive correlation, the averaged OS and LC scores exhibited a near perfect positive correlation. The differences identified between the pooled and averaged scores do appear to have been influenced by listener variability between judgments of OS and LC. Further, the acoustic models that were created in response to the spectral and temporal variations in our TE speaker samples were found to account for the variations in the listeners average LC score. Our findings of strong correlations between OS and LC suggest that the use of LC as an auditory-perceptual dimension in those who rely on TE voice and speech may be of clinical utility in documenting postlaryngectomy voice/speech outcomes.

## Data Availability

Data is contained within the article.

## References

[1] Singer MI; Blom ED An endoscopic technique for restoration of voice after laryngectomy. Ann. Otol. Rhinol. Laryngol 1980, 89, 529–533. 7458140 10.1177/000348948008900608

[2] Singer MI Tracheoesophageal speech: Vocal rehabilitation after total laryngectomy. Laryngoscope 1983, 93, 1454–1465. 6633118

[3] Singer MI; Blom ED; Hamaker RC Voice rehabilitation after total laryngectomy. J. Otolaryngol 1983, 12, 329–334. 6644863

[4] Hilgers FJ; Balm AJ Long-term results of vocal rehabilitation after total laryngectomy with the low-resistance, indwelling Provox voice prosthesis system. Clin. Otolaryngol. Allied Sci 1993, 18, 517–523. 8877233 10.1111/j.1365-2273.1993.tb00627.x

[5] van Sluis KA; van der Mole L; van Son RJJH; Hilgers FJ; Bhairosing PA; van den Brekel MEM Objective and subjective voice outcomes after total laryngectomy: A systematic review. Eur. Arch. Otorhinolaryngol 2018, 275, 11–26. 29086803 10.1007/s00405-017-4790-6 PMC5754416

[6] Robbins J; Fisher H; Blom E; Singer MI A comparative acoustic study of normal, esophageal, and tracheoesophageal speech production. J. Speech Hear. Dis 1984, 49, 202–210. 10.1044/jshd.4902.202 6716991

[7] Xi S Effectiveness of voice rehabilitation on vocalisation in postlaryngectomy patients: A systematic review. Int. J. Evid. Based Healthcare 2010, 8, 256–268. 10.1111/j.1744-1609.2010.00177.x 21091891

[8] Clements K; Rassekh C; Seikaly H; Hokanson J; Calhoun K Communication after laryngectomy: An assessment of patient satisfaction. Arch. Otolaryngol. Head Neck Surg 1997, 123, 493–496. 9158395 10.1001/archotol.1997.01900050039004

[9] Williams S; Watson J Speaking proficiency variations according to method of alaryngeal voicing. Laryngoscope 1987, 97, 937–939. 3586817

[10] Eadie TL; Bowker BC Coping and quality of life after total laryngectomy. Otolaryngol. Head Neck Surg 2012, 146, 959–965. 22307574 10.1177/0194599812437315 PMC3360982

[11] Stewart MG; Chen AY; Stach CB Outcomes analysis of voice and quality of life in patients with laryngeal cancer. Arch. Otolaryngol. Head Neck Surg 1998, 124, 143–148. 9485104 10.1001/archotol.124.2.143

[12] Eadie TL; Doyle PC Auditory-perceptual scaling and quality of life in tracheoesophageal speakers. Laryngoscope 2004, 114, 753–759. 15064636 10.1097/00005537-200404000-00030

[13] Robbins J Acoustic differentiation of laryngeal, esophageal, and tracheoesophageal speech. J. Speech Lang. Hear. Res 1984, 27, 577–585. 10.1044/jshr.2704.577 6521466

[14] Kotby MN; Hegazi MA; Kamal I; El Dien G; Nassar J Aerodynamics of the pseudoglottis. Folia Phoniat. Logopaed 2009, 61, 24–28. 10.1159/000188660 19129709

[15] Weinberg B; Moon JB Airway resistances of Blom-Singer and Panje low pressure tracheoesophageal puncture prostheses. J. Speech Hear. Res 1986, 51, 169–172. 10.1044/jshd.5102.169 3702364

[16] Moon JB; Weinberg B Aerodynamic and myoelastic contributions to tracheoesophageal voice production. J. Speech Lang. Hear. Res 1987, 30, 387–395. 10.1044/jshr.3003.387 3669645

[17] Tardy-Mitzell A; Andrews ML; Bowman SA Acceptability and intelligibility of tracheoesophageal speech. Arch. Otolaryngol 1985, 111, 213–215. 3977751 10.1001/archotol.1985.00800060037002

[18] Pindzola RH; Cain BH Acceptability ratings of tracheoesophageal speech. Laryngoscope 1988, 98, 394–397. 3352438 10.1288/00005537-198804000-00007

[19] Eadie TL; Doyle PC Direct magnitude estimation and interval scaling of naturalness and severity in tracheoesophageal speakers. J. Speech Lang. Hear. Res 2002, 45, 1088–1096. 12546479 10.1044/1092-4388(2002/087)

[20] Eadie TL Quality of Life after Total Laryngectomy. In Invitational Round Table “Evidence-Based Voice and Speech Rehabilitation in Head and Neck Cancer”; 2008; p. 61. Available online: https://www.researchgate.net/profile/Michiel-Van-Den-Brekel/publication/254919115_Proceedings_Invitational_Round_Table_Evidence-based_Voice_and_Speech_Rehabilitation_in_Head_and_Neck_Cancer_May_15-16_2008_Amsterdam/links/53f21dc20cf272810e4c9702/Proceedings-Invitational-Round-Table-Evidence-based-Voice-and-Speech-Rehabilitation-in-Head-and-Neck-Cancer-May-15-16-2008-Amsterdam.pdf (accessed on 15 December 2023).

[21] Baggs TW; Pine SJ Acoustic characteristics: Tracheoesophageal speech. J. Commun. Dis 1983, 16, 299–307. 10.1016/0021-9924(83)90014-x 6571180

[22] van As-Brooks CJ; Koopmans-van Beinum FJ; Pols LC; Hilgers FJ Acoustic signal typing for evaluation of voice quality in tracheoesophageal speech. J. Voice 2006, 20, 355–368. 16185840 10.1016/j.jvoice.2005.04.008

[23] D’Alatri L; Bussu F; Scarano E; Paludetti G; Marchese MR Objective and subjective assessment of tracheoesophageal prostheis voice outcome. J. Voice 2012, 26, 607–613. 22209062 10.1016/j.jvoice.2011.08.013

[24] Bocklet T; Riedhammer K; Nöth E; Eysholdt U; Haderlein T Automatic intelligibility assessment of speakers after laryngeal cancer by means of acoustic modeling. J. Voice 2012, 26, 390–397. 21820272 10.1016/j.jvoice.2011.04.010

[25] Kreiman J; Gerratt BR; Kempster GB; Erman A; Berke GS Perceptual evaluation of voice quality: Review, tutorial, and a framework for future research. J. Speech Hear. Res 1993, 36, 21–40. 8450660 10.1044/jshr.3601.21

[26] Stevens SS Psychophysics: Introduction to Its Perceptual, Neural and Social Prospects; Riley: New York, NY, USA, 1975.

[27] Fairbanks G Voice and Articulation Drillbook, 2nd ed.; Harper and Row: New York, NY, USA, 1960.

[28] O’Brian S; Packman A; Onslow M; Cream A; O’Brian N; Bastock K Is listener comfort a viable construct in stuttering research? J. Speech Lang. Hear. Res 2003, 46, 503–509. 14700389 10.1044/1092-4388(2003/041)

[29] Ghasemzadeh H; Doyle PC; Searl J Image representation of the acoustic signal: An effective tool for modeling spectral and temporal dynamics of connected speech. J. Acoust. Soc. Am 2022, 152, 580–590. 35931551 10.1121/10.0012734 PMC9458292

[30] Bellandese MH; Lerman JW; Gilbert HR An acoustic analysis of excellent female esophageal, tracheoesophageal, and laryngeal speakers. J. Speech Lang. Hear. Res 2001, 44, 1315–1320. 11776367 10.1044/1092-4388(2001/102)

[31] Eadie TL; Doyle PC; Hansen K; Beaudin PG Influence of speaker gender on listener judgments of tracheoesophageal speech. J. Voice 2008, 22, 43–57. 17055223 10.1016/j.jvoice.2006.08.008

[32] Susca M; Healey EC Perceptions of simulated stuttering and fluency. J. Speech Lang. Hear. Res 2001, 44, 61–72. 11218110 10.1044/1092-4388(2001/006)

[33] Bickford J; Coveney J; Baker J; Hersh D Living with the altered self: A qualitative study of life after total laryngectomy. Int. J. Speech-Lang. Path 2013, 15, 324–333. 23586580 10.3109/17549507.2013.785591

[34] Lundström E; Hammarberg B Speech and voice after laryngectomy: Perceptual and acoustical analyses of tracheoesophageal speech related to voice handicap index. Folia Phoniat. Logopaed 2011, 63, 98–108. 10.1159/000319740 20938189

[35] Doyle PC; Swift ER; Haaf RG Effects of listener sophistication on judgments of tracheoesophageal talker intelligibility. J. Commun. Dis 1989, 22, 105–113. 10.1016/0021-9924(89)90027-0 2723141

[36] Searl JP; Small LH Gender and masculinity–femininity ratings of tracheoesophageal speech. J. Commun. Dis 2002, 35, 407–420. 10.1016/s0021-9924(02)00092-8 12194562

[37] Trudeau MD; Qi Y Acoustic characteristics of female tracheoesophageal speech. J. Speech Hear. Dis 1990, 55, 244–250. 10.1044/jshd.5502.244 2329786

[38] Leemans M; van Sluis KE; van Son RJ; van den Brekel MW Interaction of functional and participation issues on quality of life after total laryngectomy. Laryngoscope Investig. Otolaryngol 2020, 5, 453–460. 10.1002/lio2.381 PMC7314459 32596487

[39] van Sluis KE; Kornman AF; van der Molen L; van den Brekel MW; Yaron G Women’s perspective on life after total laryngectomy: A qualitative study. Int. J. Lang. Commun. Dis 2020, 55, 188–199. 10.1111/1460-6984.12511 PMC7079180 31674722

[40] Bickford JM; Coveney J; Baker J; Hersh D Self-expression and identity after total laryngectomy: Implications for support. Psycho-Oncology 2018, 27, 2638–2644. 29927018 10.1002/pon.4818

